# Polyaniline-coated nanoparticles of zinc oxide and copper oxide as antifungal agents against *Aspergillus parasiticus*

**DOI:** 10.3389/fpls.2022.925451

**Published:** 2022-09-29

**Authors:** Younas Sohail, Nadeem Raza, Nasir Shakeel, Hina Raza, Suryyia Manzoor, Ghazala Yasmin, Amjad Iqbal, Shamaila Manzoor, Munirah D. Albaqami, Saikh Mohammad Wabaidur

**Affiliations:** ^1^Department of Botany, Emerson University Multan, Multan, Pakistan; ^2^Department of Chemistry, Emerson University Multan, Multan, Pakistan; ^3^Faculty of Chemistry, Silesian University of Technology, Gilwice, Poland; ^4^Faculty of Pharmacy, Bahauddin Zakariya University, Multan, Pakistan; ^5^Faculty of Science, Institute of Chemical Sciences, Bahauddin Zakariya University, Multan, Pakistan; ^6^Silesian University of Technology, Gilwice, Poland; ^7^Dipartimento di Fisica e Astronomia and LENS, Università degli Studi di Firenze, Sesto Fiorentino, Italy; ^8^Department of Chemistry, College of Science, King Saud University, Riyadh, Saudi Arabia

**Keywords:** antifungal efficiency, *Aspergillus parasiticus*, green synthesis, *Manilkara zapota*, metallic oxide nanoparticles, polyaniline

## Abstract

*Aspergillus parasiticus (A. parasiticus)* is known for producing aflatoxins and is a major threat to the food industry. Green synthesis of nanoparticles (NPs) is a cost-effective and environment-friendly approach. A variety of NPs have been explored as antifungal agents; however, their antifungal characteristics need to be further enhanced to compete with traditional fungicides. The present work describes the green synthesis of ZnO and CuO NPs by precipitation method using aqueous leaf extract of *Manilkara zapota* and their surface modification through polyaniline (PANI). Still, there is no published study on the application of PANI-coated particles as antifungal agents against *A. parasiticus and hence* was the focus of this work. The polymer-coated NPs were synthesized, characterized, and investigated for their antifungal properties against *A. parasiticus*. Textural and structural characterization of PANI-coated and non-coated ZnO and CuO NPs were confirmed through FT-IR, SEM, and XRD techniques. The PANI-coated NPs presented higher fungal growth inhibition (%) as compared to the non-coated ones. The maximum inhibition of 77 ± 2% (*n* = 3) was shown by PANI/ZnO NPs at a concentration of 12 mmol L^−1^ and 72 h of incubation. The non-coated NPs presented a lower inhibition rate with respect to their coated NPs, thus justifying the role of polymeric coating in improving antifungal efficiency.

## Introduction

Aflatoxins are mycotoxins mainly produced by two fungal species *Aspergillus parasiticus (A. parasiticus)* and *Aspergillus flavus*, common opportunistic pathogens, and saprophytes. These pathogens contaminate wheat, maize, rice, cottonseed, pistachios, groundnuts, Brazil nuts, spices, and copra in tropical and semi-tropical areas (Shephard, 2003). These fungal species attack susceptible food crops during the pre-harvesting, processing, and storage of crops (Diener et al., 1987). Some of the *A. flavus* isolates do not release aflatoxins, while about all isolates of *A. parasiticus re*lease aflatoxins (Klich and Pitt, 1988). Aflatoxins of *A. parasiticus* are the most potent naturally occurring carcinogens in humans and animals. *A. parasiticus* release more than 90% of aflatoxins from cells through specialized vesicles called aflatoxisomes which play an important role in the synthesis and export of aflatoxins (Chanda et al., 2009, 2010).

Various approaches have been applied to control aflatoxins producing fungi but the use of nanoparticles (NPs) as antifungal agents is a cost-effective and environment-friendly approach. Enhanced thermal conductivity, catalytic activity, chemical steadiness, non-linear optical performance, and strong adsorption capability of NPs significantly increase their performance and applications (Das et al., 2018; Kabir et al., 2019). Multiple NPs have been applied against microorganisms including bacteria and fungi. For instance, NPs of Ag have been checked as antibacterial, antiseptic, antimicrobial, antiviral, antifungal, and antitoxic agents (Zinjarde, 2012; Salem and Fouda, 2021). Similarly, copper oxide (CuO) NPs have also been investigated as antimicrobial, anticancer, and antioxidant (Mahapatra et al., 2008; Maqbool et al., 2017; Mohammed et al., 2018). Zinc oxide (ZnO) NPs are self-cleansing, non-toxic (Yadav et al., 2006), suitable for skin, antimicrobial, and UV-blocker in sunscreens (Krishnan and Pradeep, 2009). ZnO NPs have also been used in tissue regeneration, bioimaging, implant coatings, wound healing, and cancer therapies (Oliveira and Zarbin, 2005; Mirzaei and Darroudi, 2017; Mishra et al., 2017; Ullah et al., 2017; Iqbal et al., 2019). They have further been proved effective in enhancing ruminal fermentation aiding in the production of methane gas (Palangi et al., 2022). NPs prepared by plants are more effective against microbes than those prepared through chemical approaches, for example, NPs prepared through extracts of *Ocimum tenuiflorum* and *Azadirachta indica* exhibit health-improving characteristics (Ramteke et al., 2013; Verma and Mehata, 2016).

Physical and chemical approaches to NPs preparation are labor intensive, expensive, and release hazardous chemicals into the environment (Manzoor et al., 2021). So, researchers are shifting quickly toward the green synthesis of metal and metal oxide NPs (Pugazhendhi et al., 2018). Various microorganisms (such as bacteria, algae, fungi, and yeast) and plant extracts are used as reducing agents for the green synthesis of metal and metal oxide NPs (Nasrollahzadeh et al., 2019). Previously, NPs of ZnO have been synthesized through *Aloe barbadensis* (Sangeetha et al., 2011), *Aspalathus linearis* (Diallo et al., 2015), *Calotropis gigantea* (Vidya et al., 2013), and *Hibiscus sabdariffa* (Soto-Robles et al., 2019). Whereas, CuO NPs have been synthesized from *Gloriosa superba* (Naika et al., 2015), *Allium sativum* (Velsankar et al., 2020), *Calotropis gigantea* (Sharma et al., 2015), *Vitis vinifera* (Gultekin et al., 2017), and *Pterospermum acerifolium* (Saif et al., 2016). Unique characteristics can be inherited by the NPS when synthesized using different plant extracts. In this aspect, *Manilkara zapota* leaf extract has been proven to possess antimicrobial activities with the potential of further exploring its specific properties. Its extract can be evaluated to synthesize NPS. This is possible due to the fact that *M. zapota* contains phytochemicals that could serve as reducing agents and particle stabilizers during the synthesis of nanoparticles. The major phytochemicals present in its leaves are lupeol acetate, flavonoids, oleanolic acid, glycosides, apigenin-7-O-α-L-rhamnoside, myricetin-3-O-α-L-rhamnoside, terpenoids, and caffeic acid with different applications (Ma et al., 2003).

Certain polymeric coatings, such as polyaniline (PANI), chitosan, and so on, on green-synthesized NPS can aid in their antimicrobial ability (Pinho and Piedade, 2020; Manzoor et al., 2021). Polymers having antimicrobial potential play important role in the fields of food packaging, pharmacy, health care, and tissue implants (Jain et al., 2014). Owing to their biocompatibility and low cytotoxicity, they have been potentially used in biomedicine (Saikia et al., 2010). PANI is novel because of its easy synthesis, electroactivity, and doping–dedoping chemistry (Syed and Dinesan, 1991). It has been widely applied in the field of neural probes, biosensors, tissue engineering materials, and controlled drug delivery systems (Balint et al., 2014). Moreover, it has significant antibacterial properties, especially PANI and PANI/metal NPs are effective against *E. coli* and *Staphylococcus aureus* bacteria (Dhivya et al., 2019). Furthermore, the literature on green synthesized and PANI-coated metal oxide NPs and their applications toward antifungal efficiency is very scarce. The present research was organized to produce metallic oxide nanoparticles (NPs) of CuO and ZnO through leaf extract of *M. zapota*. To improve the antifungal efficiency of metal oxide NPs, we coated green synthesized NPs of CuO and ZnO with PANI. Both PANI-coated and non-coated NPs were checked as antifungal agents against *A. parasiticus*, a cancer-causing fungus.

## Materials and methods

Copper acetate (99%), zinc nitrate (99%), ammonium peroxydisulphate (98%), acetone (99.5%), HCl (37%), aniline (99.5%), ethanol (99.8%), methanol (99%), agar (98%), and glucose (98%) were purchased from Riedel-de Haen (Seelze, Germany). Streptomycin (99.6%) was obtained from Sigma Aldrich (St. Louis, MO, USA). *A. Parasiticus* was obtained from Punjab university culture bank FCBP Acc. No FCBP-PTF1227, Source of isolation: Chickpea seeds, Lahore, Pakistan. FCBP-DNA No. 1227, Preservation Condition Temp. 04°C, MEA slants. SPSS version 26.0 was employed for statistical analysis.

### Green synthesis of CuO and ZnO nanoparticles

Green synthesis of CuO and ZnO NPs was achieved using leaf extract of *M. zapota* locally known as Chikoo. About 400-g leaves of *M. zapota* were washed with deionized water and cut into small pieces with a sterilized knife. All the pieces of leaves were boiled in 400 mL of deionized water for 60 min until the color of the water gets slightly amber. After cooling at room temperature, the extract was filtered and stored in the refrigerator for further use.

For the preparation of CuO NPs, 50 mL of 50 mg L^−1^ copper acetate solution was mixed with 150 mL leaf extract at 60°C in a beaker. The temperature was further raised to 80°C with continuous stirring for 4 h. The particles were formed by turning the solution turbid, which was filtered and washed three times with deionized water. These particles were heated in a furnace at 500°C for 1 h, then cooled to room temperature and stored for further tests.

A similar procedure was followed for the preparation of ZnO NPs by the addition of 50 mL of 50 mg L^−1^ zinc nitrate solution in 150 mL leaf extract of *M. zapota* at 60°C. The solution was boiled until precipitates were obtained. These particles were calcined in the furnace for 2 h at 400°C.

### Coating of CuO and ZnO NPs with polyaniline

About 0.2 M solution of each aniline hydrochloride (C_6_H_8_ClN) and ammonium peroxydisulphate (H_8_N_2_O_8_S_2_) were prepared. A 10 mL of 0.2 M aniline monomer and 20 mL of 0.1 M HCl were mixed in a beaker. To this solution, 0.75 g of CuO NPs was added with continuous stirring. Then, 0.25 M Ammonium peroxydisulphate was added dropwise to the above solution as an oxidizing agent with continuous stirring for 24 h. Afterward, coated particles were filtered and washed with deionized H_2_O followed by 0.1 M HCl and then with acetone many times to obtain a clear filtrate. In the end, particles were dried at 70°C in an oven for 24 h. Dried precipitates were ground to make powder for further characterization.

A similar procedure was adopted for coating zinc oxide NPs with polyaniline by the addition of 0.75 g NPs of ZnO with continuous sonication. After polymerization, the solution changed its color from sky-blue to deep-green. The solution was kept with stirring for 24 h and filtered. The filtrate was washed with deionized water, followed by 0.1 M HCl and then with acetone. The coated particles were dried in an oven at 80°C for 24 h.

### Characterization of coated and non-coated NPs

#### Scanning electron microscopy

The surface morphology of NPs was observed through a scanning electron microscope (SEM, LEO-1530, Oberkochen, Germany) under high vacuum conditions in a series of magnification ranges. NPs were spread in ethanol, and a drop was placed on a carbon-coated copper sample holder. The ethanol was allowed to evaporate completely before SEM analysis.

#### FTIR studies

Bruker Alpha II spectrometer (Billerica, MA, USA) with a single reflection diamond ATR module was used for FTIR spectra of NPs. The scanning was performed between 4,000 and 650 cm^−1^.

#### X-ray diffraction analysis

The crystallinity of the NPs was studied through x-ray diffraction (XRD) analysis. Bruker D8 (Karlsruhe, Germany) was used to obtain XRD spectra of NPs.

### Anti-fungal activity of coated and non-coated NPs

The activity of NPs as antifungal agents was evaluated against *A. parasiticus* by measuring the mycelial radial growth in NPs' incorporated medium. The whole apparatus (Petri plates, pipettes, and micropipettes) and glass wares were autoclaved before use. Potato Dextrose Agar (PDA) medium was prepared by mixing 25 g of dextrose and 25 g of agar in 500 g of boiled and mashed potatoes. Afterward, 0.5 g of streptomycin sulfate was added to the PDA medium to prevent bacterial growth. In the next step, suspensions of PANI coated and non-coated NPs of CuO and ZnO with four different concentrations (3, 6, 12, and 24 mmol L^−1^) were prepared in an aqueous medium and added to the flasks containing PDA. The petri dishes were then prepared using the PDA media with various concentrations of NPs under investigation. Suspension of *A. Parasiticus* was prepared and placed in a well-created in the center of a petri dish containing PDA. One petri dish was used as control which did not contain any NP. All the petri plates were incubated at 25°C and mycelial radial growth were measured after 72 h in cm.

## Results and discussion

**Figure 1:**
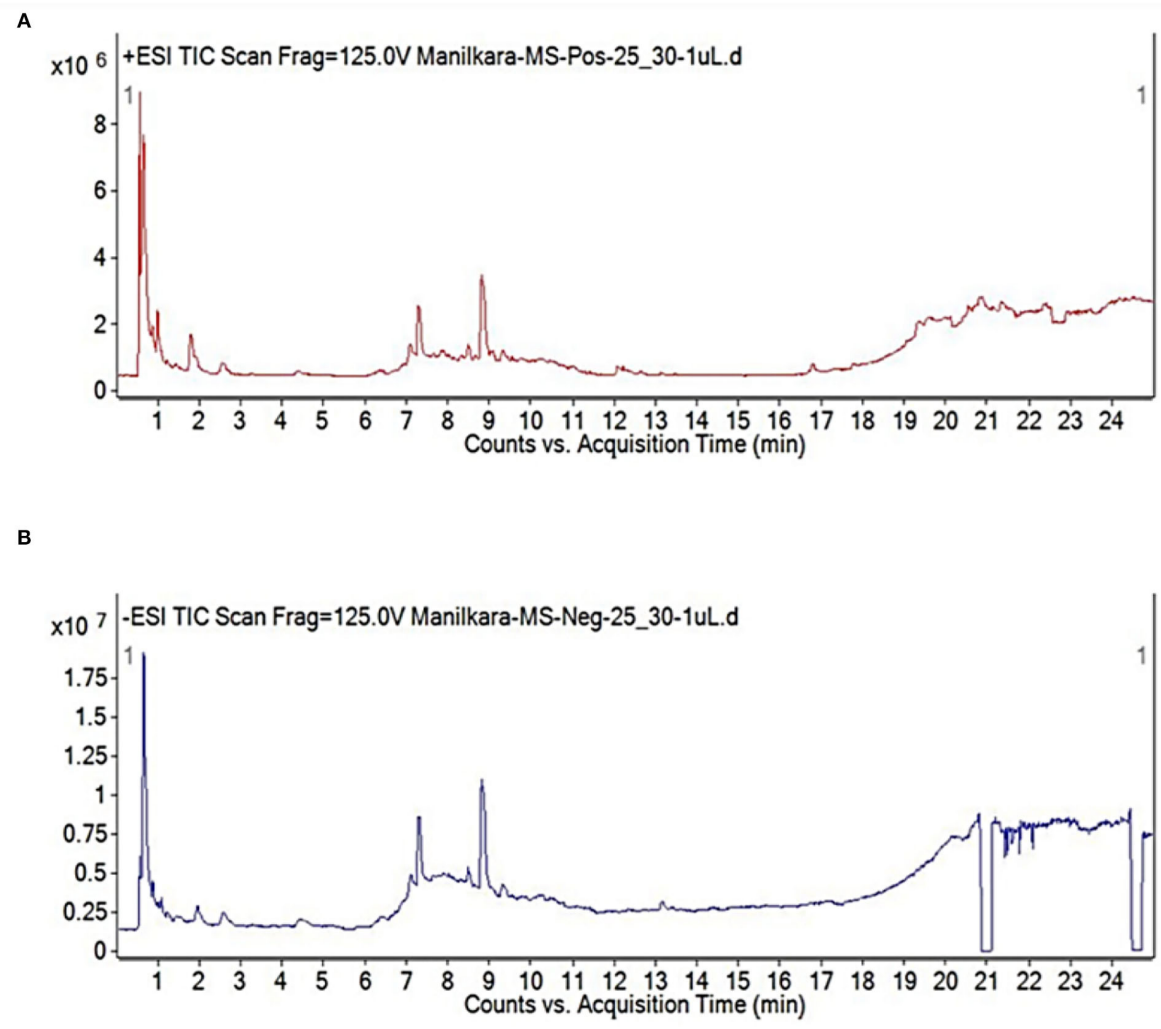
Chromatogram of aqueous leaf extract by LC-QTOF MS in **(A)** positive and **(B)** negative ionization modes (Tamsir et al., 2020).

**Figure 2:**
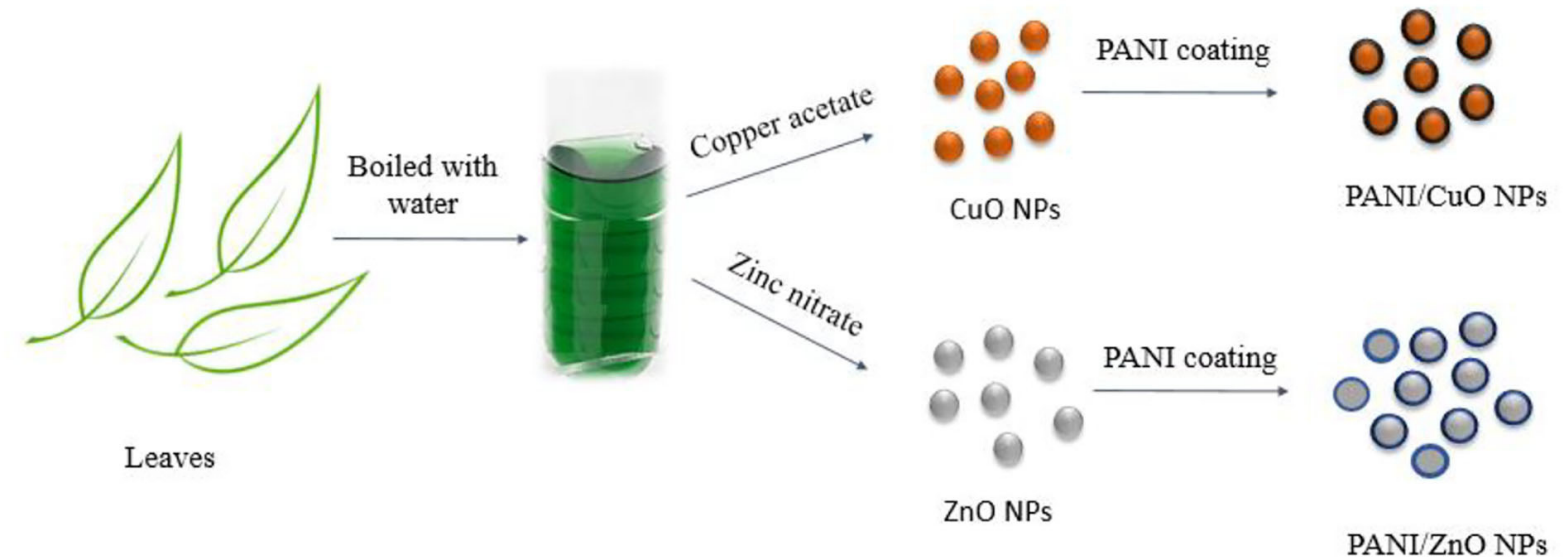
Schematic representation of NPs' synthesis.

Various phytochemicals that constitute the essential part of the aqueous extract of *M. zapota*'s leaves are possibly capable of serving as reducing agents (Islam et al., 2021). Tamsir et al. identified 39 phytochemicals in the aqueous leaf extract. Some among them are isoorientin 6”-O-caffeate, S-Ribosyl-L-homocysteine, protoleucomelone, 4-(3-Pyridyl)-butanoic acid, 1-caffeoyl-beta-D-Glucose, robinetinidol-4alpha-ol, C16 sphinganine, apocynin A, nonic acid, quercitin 3-(6”-acetylglucoside), robinetinidol-4alpha-ol, quinic acid, and m-coumaric acid. The phytochemical analysis of leaf extract by liquid chromatography quadrupole time-of-flight mass spectrometry (LC-ESI-Q-TOF) given in Figure 1 confirmed the presence of these compounds (Tamsir et al., 2020). Although these phytoconstituents are meant to protect plants against infection or predation by microbes, they play a very important role in the green synthesis of NPs by causing the reduction of metal ions into metals. The synthetic scheme of NPs is given in Figure 2.

### FTIR analysis

Kiriyanthan et al. have described the presence of hydroxyl groups, C=C, C-O, and C-O-C, as major functional groups in leaf extract of *M. zapota* through the FTIR spectrum (Kiriyanthan et al., 2020).

**Figure 3:**
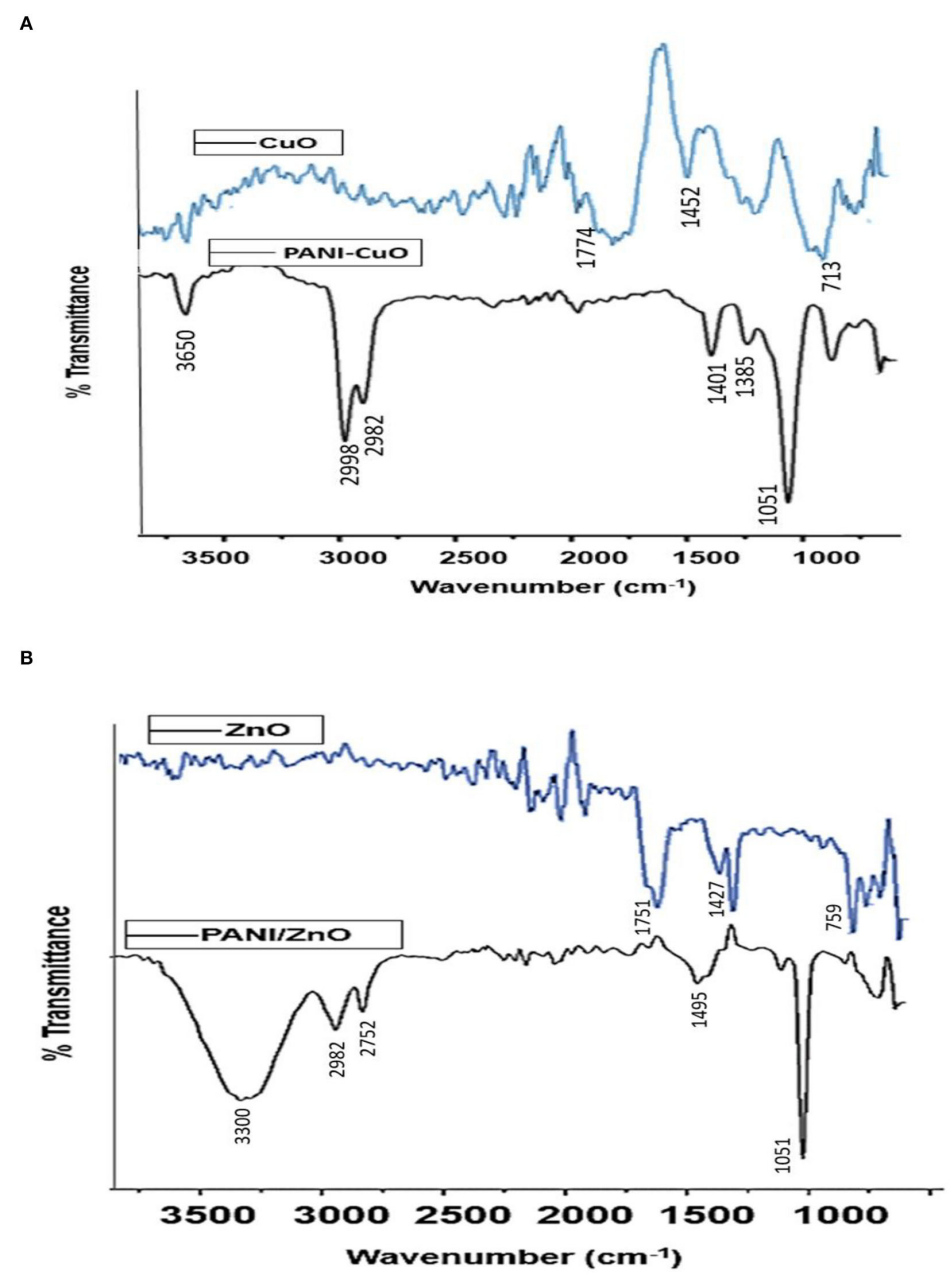
FTIR spectra of **(A)** CuO and PANI/CuO NPs and **(B)** ZnO and PANI/ZnO NPs.

The significant band at 2,020 cm^−1^ in the FTIR spectrum of CuO NPs demonstrated the presence of C=C, while the peak at 1,452 cm^−1^ indicated C-H bending vibration. The band at 1,774 cm^−1^ indicated the presence of a C=O bond and at 713 cm^−1^ demonstrated the existence of Cu-O (Figure 3A). The presence of the carbonyl and hydroxyl functional groups on the NPs' surface is most probably due to the reducing agents of aqueous leaf extract used for their synthesis. In the case of PANI/CuO NPs, a slight shift in the case of NH bond was observed. The band at 3,650 cm^−1^ is indicative of PANI's NH bond (Figure 3A) and at 1,051 cm^−1^ represented benzenoid, quinonoid rings, and aromatic C-H in-plane bending mode. The C-N stretching was observed at 1,244 cm^−1^. Emeraldine salt configuration was seen at 1,401 cm^−1^ with conducted protonated configuration along the C-N stretching mode of imine. N-H Wag stretching vibration showed the existence of amine at 887 cm^−1^. The band at 2,982 cm^−1^ demonstrated C-H stretching vibration with the presence of alkenes. The presence of these functional groups thus confirms the coating of NPs with PANI.

The FTIR spectrum of ZnO nanoparticles (Figure 3B) shows a band at 1,427 cm^−1^ assigned to C-C stretching vibrations. C=O band due to polyphenols appeared at 1,750 cm^−1^ suggesting its asymmetric vibration mode, while symmetric vibration resulted in a band at 1,385 cm^−1^. ZnO hexagonal phase in the IR spectrum is represented by the band at 759 cm^−1^. The presence of carbonyl and C-C bands in the ZnO NPs' spectrum is possibly due to the greener approach of synthesis involving the leaves extracts. PANI/ZnO NPs' FTIR spectrum is shown in Figure 3B. The band at 3,300 cm^−1^ indicates the O-H of PANI. C-H stretching vibration due to the presence of alkenes appeared at 2,982 cm^−1^. The band at 1,318 cm^−1^ is due to C-C stretching vibrations, while at 1,250 cm^−1^ suggests the existence of carbon-nitrogen stretching. A prominent band appeared at 1,051 cm^−1^ representing benzenoid and quinonoid rings. The peak of 731 cm^−1^ represented the C-H bonding mode of the aromatic ring. FTIR spectra of PANI/ZnO NPs thus provided an insight into surface functionalities and confirmed the coating of polyaniline in the nanoparticles.

### SEM analysis

**Figure 4:**
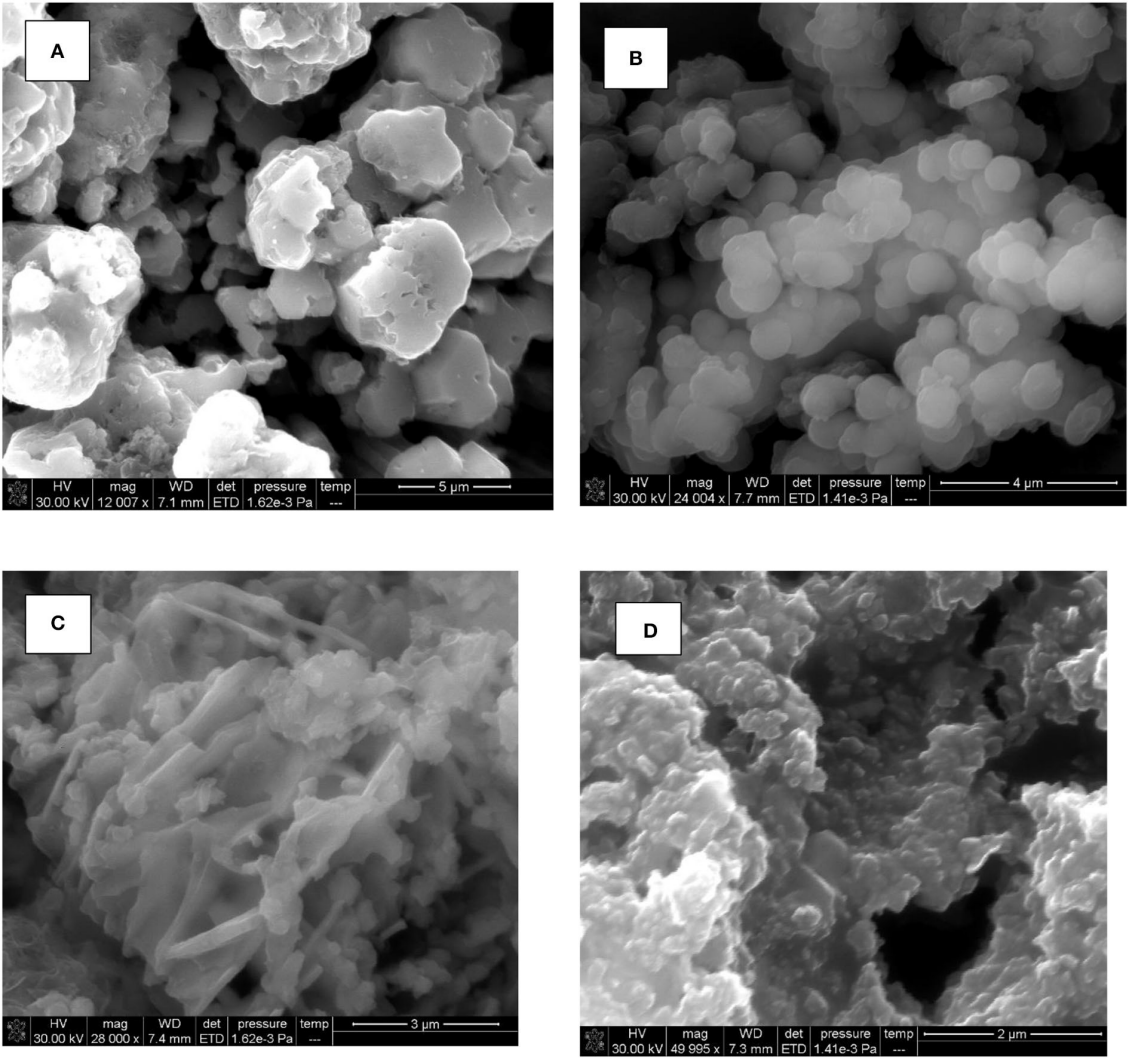
SEM images of **(A)** CuO, **(B)** PANI/CuO, **(C)** ZnO, and **(D)** PANI/ZnO.

The SEM image of CuO NPs demonstrated the existence of the individual and aggregated spherical NPs (Figure 4A). Figure 4B describes the SEM image of PANI/CuO NPs which predominantly appeared as bigger, grainy, and spherical clusters.

In the case of ZnO nanoparticles, irregular criss-cross and rod-like structures were seen (Figure 4C). While, the SEM image of PANI/ZnO NPs demonstrated the uniform spread of spherical particles (Figure 4D). Nearly spherical-structured granules were observed interlinked with each other. This change in morphology possibly occurred due to the coating with the polymer.

### XRD analysis

The crystal size of modified and unmodified NPs was determined by following the Debye Scherrer equation.


(1) $$\begin{array}{l} D\;=\;K\lambda /\beta \cos\theta \end{array}$$

Where D is the size of particle in nm, K is the correction factor dependent on crystallite's gyration radius, λ is the wavelength of X-ray radiation, β is the full width at half maximum (FWHM) in radian, and θ is the Bragg's diffraction angle.

**Figure 5:**
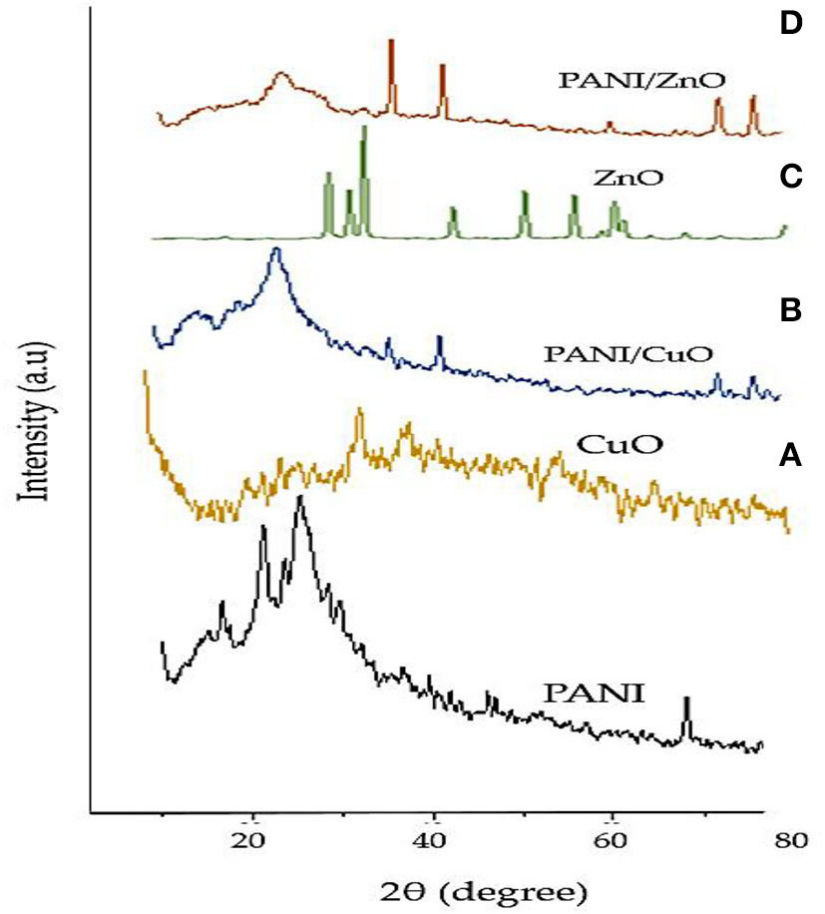
XRD spectra of **(A)** CuO, **(B)** PANI/CuO, **(C)** ZnO, and **(D)** PANI/ZnO.

The XRD spectra of polyaniline and the coated and non-coated NPs under investigation are given in Figure 5. The crystalline peaks of PANI appeared at 2θ of 9.7, 16.54, 21.35, and 25.12° corresponding to (001), (011), (020), and (200) planes.

The XRD peaks of green-synthesized CuO NPs with diffraction angle 2θ of 36.76, 48.37, 58.66, 62.01, and 73.41° represented (h, k, l) values of reflection from (111), (−202), (202), (−113), and (311) crystal planes. The XRD peaks evidently indicated the crystalline form of CuO NPs manufactured from *M. zapota* leaf extract. The position of peaks displayed the spherical structure of CuO NPs that was verified by the International Centre for Diffraction Data (ICDD) card no. 801916. The crystal size of CuO NPs was found about 25.5 nm indicating its nanocrystalline nature. Significant XRD peaks of PANI/CuO NPs with diffraction angle 2θ of 15.48, 25.73, 39.98, 46.44, and 81.77° represented (h, k, l) values of reflection from (121), (322), (100), (−202), and (−313) crystal planes. The XRD peaks evidently indicated the crystalline form of green synthesized PANI/CuO NPs. The position of peaks displayed the spherical arrangement of PANI/CuO NPs verified by the ICDD through card no. 00-001-1117. The size of PANI/CuO NPs was noted as 51.2 nm indicating its nanocrystalline nature.

The significant XRD peaks of green-synthesized ZnO NPs with diffraction angle 2θ of 31.82, 34.33, 36.49, 47.56, 57.16, 63.20, and 68.99° represented (h, k, l) values of reflection from (100), (002), (101), (102), (110), (103), and (201) crystal planes. The spherical arrangement of the ZnO NPs was verified by the Joint Committee on Powder Diffraction Standards (JCPDS) through card no. 361451 and verified the hexagonal wurtzite arrangement of nanoparticles. The crystalline size of green-synthesized ZnO NPs was recorded as 20.3 nm. The XRD peaks of green-synthesized PANI/ZnO NPs with diffraction angle 2θ of 25.81, 39.89, 46.60, 68.28, 82.14, and 86.60° represented (h, k, l) values of reflection from (200), (100), (102), (201), (104), and (202) crystal planes. Broad peak at 25.81° showed close similarity with polyaniline. ZnO NPs showed crystalline arrangement which was not specified in the case of polyaniline NPs. The amorphous character of polyaniline in the coated NPs indicated that the addition of ZnO NPs restrained the polyaniline molecular chain crystallization. The crystalline size of PANI/ZnO NPs was recorded as−48.2 nm.

### Study of the antifungal activities

A variety of NPs has been tested for their ability to inhibit fungal growth. The greater surface area of these nanomaterials makes them advantageous over other techniques of fungal growth inhibition, especially because nanomaterials are required in less quantity, can easily penetrate the cell, and are more reactive. Metallic NPs work by producing hydroxyl and superoxide radicals while reacting with oxygen or by electron/hole interactions (Agbe et al., 2019). All these species can cause cell oxidation as they possess high oxidative ability during interaction with the fungal cell (Zamperini et al., 2013). Further, the NPs possibly interact with the nucleic acids rendering them incapable of duplication as well as interfering with the membrane-bound enzymes (Sivasankar et al., 2019). With an aim to further enhance the conducting properties of NPs and to study the effects on antifungal properties, we prepared polyaniline-based CuO and ZnO NPs and compared them with their non-coated NPs. Both of these metals are among the most significant minerals utilized by plants for their growth.

All the prepared samples of coated and non-coated NPs were tested against wheat fungal pathogen *A. parasiticus*. Our results demonstrated that PANI/CuO and PANI/ZnO NPs exhibit good antifungal activity against the target organism. The percentage decrease of fungal growth was calculated by using the following formula:


$$\begin{array}{l} \text{Fungal growth inhibition }\left(\text{\%}\right)=\frac{dC-dT}{dC}\times 100 \end{array}$$

Where dC is the average diameter (cm) of fungal colonies in the control plate and dT is the diameter (cm) of fungal colonies in the dual culture plate (cm) (Gamsore et al., 2018). Distilled water without NPs was added to the control plate.

**Figure 6:**
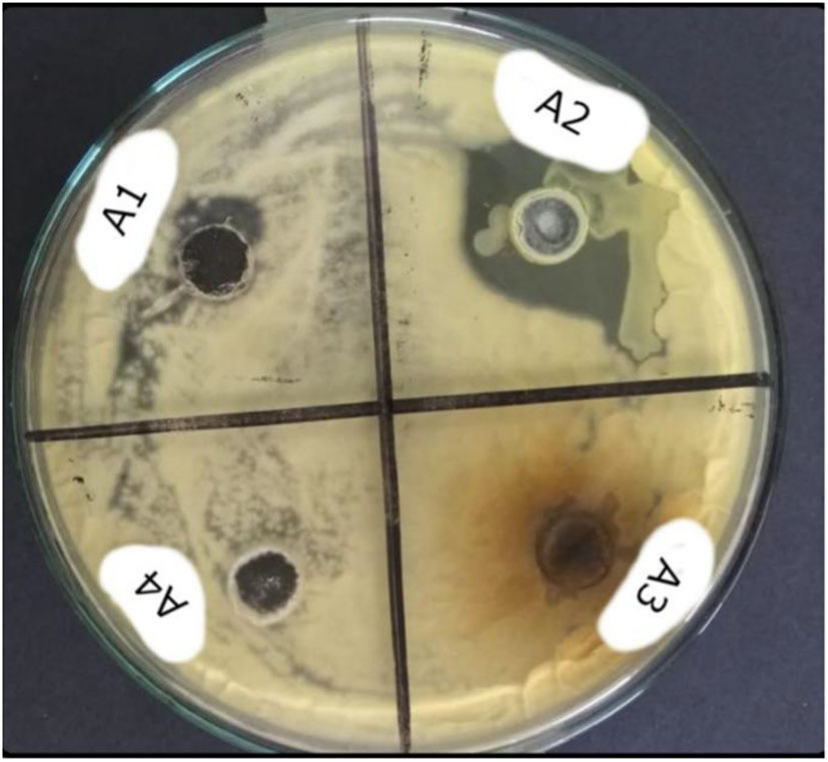
Antifungal study of PANI/ZnO (A1), ZnO (A2) PANI/CuO NPs (A3), and CuO (A4) at 12 mmol L^−1^ concentration.

The antifungal activity was determined at different concentrations ranging from 3 to 24 mmol L^−1^. However, the maximum antifungal growth inhibition occurred at 12 mmol L^−1^ (Figure 6) which might be due to the ability of fungi to form biofilms that act as barriers against antifungal agents and protect the colonies. Thus, to overcome the effect of this behavior on fungal colonies, a higher concentration of NPs is generally required (Zamperini et al., 2013). Furthermore, higher NPs concentrations could also result in greater adherence to fungal mycelia leading to the deactivation of fungal cells (Jo et al., 2009).

During the time parameter, optimization of the fungal mycelial radial growth was observed consistently for 72 h. A slow spread of fungal mycelia was noticed till 72 h, after which no further growth was detected. A further increase in concentration also had an inappreciable effect of inhibiting fungal growth. Minimum inhibitory concentration (MIC) of 3 mmol L^−1^ for PANI/ZnO and 6 mmol L^−1^ for PANI/CuO were found at which a visible inhibition of growth was observed. All the experiments were performed in triplicate and data were presented as mean value ± standard deviation. Intergroup differences were calculated by one-way analysis of variance using SPSS version 26.0. The results with *p* < 0.05 are considered statistically significant; however, the statistical analysis shows that the *p*-value in each case was higher than 0.05 suggesting that the intergroup difference was not significant (Table 1). The PANI-coated NPs presented higher fungal inhibition as compared to non-coated ones. Hence, the maximum inhibition was obtained in the case of PANI/ZnO with a percentage fungal growth inhibition value of 77%. CuO and ZnO also presented significant growth retardation, yet their antifungal properties in terms of percentage inhibition (61.5 and 62.4%, respectively) were less than their counter polymer-coated NPs.

**Table 1:** Mycelial diameter (cm) and fungal growth inhibition (%) by CuO, PANI/CuO, ZnO, and PANI/ZnO nanoparticles against *A. parasiticus* after 72 h of incubation and 12 mmol L^−1^ concentration.

**Formulation**	**Mycelial radial growth (cm)**	**Mean fungal mycelial radial growth mean±Sd**	**Fungal growth inhibition (%)**	**Mean fungal growth inhibition mean ±Sd (%)**	***p*-value**
CuO NPs	2.6	2.6 ± 2.1	61.1	61.5 ± 3.5	*p* > 0.05
	2.8		65.2		
	2.4		58.3		
PANI/CuO NPs	1.7	1.6 ± 1.4	73.6	68.5 ± 2.1	*p* > 0.05
	1.5		70.8		
	1.8		69.4		
ZnO NPs	3.1	2.7 ± 1.7	62.5	62.4 ± 1.4	*p* > 0.05
	2.6		61.1		
	2.5		63.8		
PANI/ZnO NPs	2.4	2.1 ± 0.99	79.1	77.4 ± 2.5	*p* > 0.05
	2.1		77.5		
	1.9		75.7		

### Performance evaluation

Few silver-based NPs have been described in the literature that presented inhibitory activities against *A. parasiticus*. For example, an inhibitory concentration of 8 μg mL^−1^ of AgNPs obtained from biogenic fungus was sufficient to stop the fungus mycelial growth (Bocate et al., 2019). Similarly, Ag/Zn NPs have also been synthesized and used in the treatment of this fungus. The Ag/Zn NPs required a concentration of 1,250 μg mL^−1^ to inhibit the fungal growth with a colony diameter of 1.8 cm (Sedaghati et al., 2018). They also investigated the role of different culture media (i.e., potato dextrose agar and Czapeck Dox agar) in the inhibitory effect of Ag/Zn NPs. The results showed that the growth media had no significant effect on the mycelial growth retardation of *A. parasiticus*.

The presence of the limited number of metal oxide NPs as fungicides against *A. parasiticus* thus demands further exploration of diverse types of NPs in this regard. The use of CuO- and ZnO-based NPs in this study might also be useful as important micronutrients for plants along with their antifungal abilities, resulting in their biofortification (Zafar et al., 2020).

## Conclusion

Nanoparticles can be synthesized through routinely used physical and chemicals methods but green synthesis of NPs is economically sound and environmentally friendly. The synthesis of ZnO and CuO NPs was achieved through the precipitation method using aqueous leaf extract of *M. zapota* and their surface modification was done using polyaniline. Antifungal characteristics of coated and non-coated CuO and ZnO NPs were evaluated against a threatening fungal strain: *A. parasiticus*. Although PANI/CuO presented good growth retardation (71%) in fungal mycelia, the PANI/ZnO NPs superseded their efficiency and presented a value of 77% which was the highest among all the studied NPs. The maximum retardation in fungal growth was found at 12 mmol L^−1^ and minimum inhibitory concentrations of 3 and 6 mmol L^−1^ were obtained for PANI/ZnO and PANI/CuO, respectively. Overall, the results confirmed the antifungal effects of all formulations but a non-significant difference was observed based on the *p*-value. The obtained data thus confirms the role of polyaniline in improving the antifungal properties of NPs.

## Data availability statement

The original contributions presented in the study are included in the article/supplementary material, further inquiries can be directed to the corresponding author/s.

## Author contributions

GY and SuM: conceptualization and formal analysis. GY and AI: methodology. ShM, HR, NR, NS, and YS: data interpretation. AI and SuM: investigation. MA and SMW: resources. YS, ShM, NR, and NS: writing original draft preparation. ShM, HR, YS, NS, MA, and SMW: writing review and editing. GY, MA, AI, and SMW: visualization. SuM, GY, and NR: supervision. All authors contributed to the article and approved the submitted version.

## Funding

This work was funded by the Researchers Supporting Project Number (RSP-2021/267), King Saud University, Riyadh, Saudi Arabia. The authors acknowledge the TU-Wien University Library for financial support through its Open Access Funding Program.

## Conflict of interest

The authors declare that the research was conducted in the absence of any commercial or financial relationships that could be construed as a potential conflict of interest.

## Publisher's note

All claims expressed in this article are solely those of the authors and do not necessarily represent those of their affiliated organizations, or those of the publisher, the editors and the reviewers. Any product that may be evaluated in this article, or claim that may be made by its manufacturer, is not guaranteed or endorsed by the publisher.
